# A novel histone deacetylase inhibitor, CKD5, has potent anti-cancer effects in glioblastoma

**DOI:** 10.18632/oncotarget.13265

**Published:** 2016-11-10

**Authors:** Seung Ah Choi, Pil Ae Kwak, Chul-Kee Park, Kyu-Chang Wang, Ji Hoon Phi, Ji Yeoun Lee, Chang Sik Lee, Ju-Hee Lee, Seung-Ki Kim

**Affiliations:** ^1^ Division of Pediatric Neurosurgery, Pediatric Clinical Neuroscience Center, Seoul National University Children's Hospital, Seoul, Korea; ^2^ Adolescent Cancer Center, Seoul National University Cancer Hospital, Seoul, Korea; ^3^ Department of Neurosurgery, Seoul National University Hospital, Seoul, Korea; ^4^ Department of Anatomy, Seoul National University Hospital, Seoul, Korea; ^5^ Chong Kun Dang Research Institute, CKD Pharmaceuticals, Gyeonggi-do, Korea

**Keywords:** epigenetics, histone deacetylase inhibitor, glioblastoma

## Abstract

There have been extensive efforts to improve the outcome of glioblastoma, but the prognosis of this disease has not been significantly altered to date. Histone deacetylase inhibitors (HDACIs) have been evaluated as promising anti-cancer drugs and regulate cell growth, cell cycle arrest and apoptosis in glioblastoma. Here, we demonstrated the therapeutic efficacy of a novel pan-HDACI, 7-ureido-N-hydroxyheptanamide derivative (CKD5), compared with traditional pan-HDACIs, such as suberoylanilide hydroxamic acid (SAHA) and trichostatin A (TSA), *in vitro* and *in vivo*. Compared with SAHA and TSA, CKD5 had improved cytotoxic effects and induced apoptosis, anti-proliferative activity and cell cycle arrest at G2/M phase. Furthermore, CKD5 significantly reduced tumor volume and prolonged the survival *in vivo* compared with TSA, suggesting improved anti-cancer efficacy among HDACIs. Our results demonstrate that the novel HDACI CKD5 is a promising therapeutic candidate for glioblastoma.

## INTRODUCTION

Glioblastoma is one of the most aggressive cancers and is often resistant to conventional anti-cancer treatments [1, 2]. The current standard treatment involves maximal surgical resection and concomitant chemo-radiotherapy followed by adjuvant chemotherapy with temozolomide [3]. However, the prognosis is still poor due to the refractoriness of glioblastoma to the various secondary treatments as well as the standard management [2, 4]. Therefore, the development of new therapeutic targets is urgently needed.

Cancer development is mediated by both aberrant genetic and epigenetic alterations [5]. Histone deacetylases (HDACs) are epigenetic regulators of gene expression that remove the acetyl group from histones, which is often associated with gene repression. Overexpression of HDACs in various types of cancer provides a rationale for targeting these enzymes in cancer [6–10]. Recently, many studies reported increased expression of HDACs in brain malignancies, including glioblastoma, among which HDAC4 (class IIA) is the most important target [8]. Previous publications confirmed the efficacy of HDAC inhibitors (HDACIs) as anti-cancer drugs or radiosensitizers in pre-clinical studies for glioblastoma, and several clinical trials have already been launched [8, 11–15]. For the anti-cancer effect against glioblastomas, cell cycle regulation is believed to be a key mechanism of HDACIs [16]. With the increasing focus on HDACIs, SAHA and TSA have been assessed in the treatment of many different types of cancer [17–19]. However, low doses of SAHA and TSA were not effective anti-cancer agents, and high doses had strong neurotoxic effects [20–23].

In the present study, we evaluated the anti-cancer effects of a novel pan-HDACI, 7-ureido-N-hydroxyheptanamide derivative (CKD5), on glioblastoma cell lines and patient-derived cells. CKD5 showed more potent cytotoxic effects than suberoylanilide hydroxamic acid (SAHA) and trichostatin A (TSA) *in vitro* and superior therapeutic efficacy compared to SA *in vivo*.

Our study suggests that CKD5 might be a potent therapeutic agent for the treatment of glioblastoma.

## RESULTS

### CKD5 is a potent cytotoxic and cytostatic agent in glioblastoma cells

**Figure 1:**
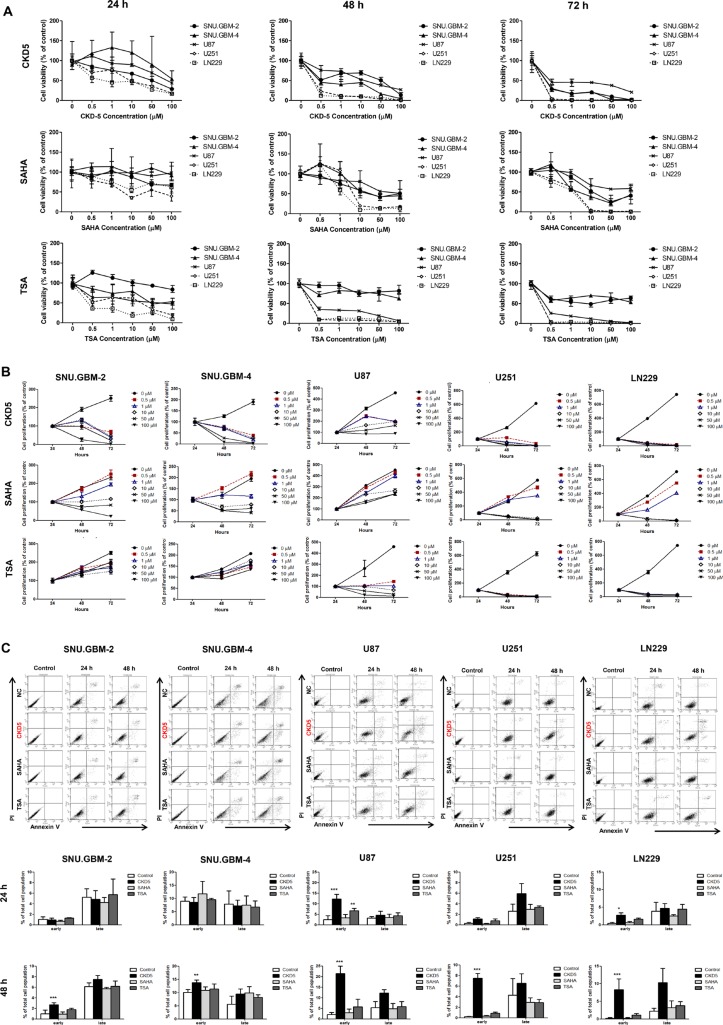
Cell viability, proliferation and apoptosis analyses (**A**) The cell viability and (**B**) proliferation of glioblastoma cells treated with different concentrations of histone deacetylase inhibitors (HDACIs) at 24, 48 and 72 h. CKD5, SAHA and TSA inhibited the cell growth of glioblastoma. Following HDACIs treatment, the cell viability and proliferation were decreased. CKD5 is the most effective at reducing tumor cell growth. (**C**) Induction of apoptosis in glioblastoma cells. Early apoptosis was significantly increased by CKD5 compared to SAHA and TSA. *p < 0.05, **p < 0.01, ***p < 0.005.

To compare the anti-cancer effects of the HDACIs (CKD5, SAHA and TSA) in glioblastoma cells, cell viability and proliferation assays were performed. All HDACIs suppressed the cell growth in a dose-dependent manner at 72 h (Figure 1A). The IC_50_ values of the HDACIs were calculated. Importantly, patient-derived glioblastoma cells were more sensitive to CKD5 than SAHA or TSA (IC_50_ of CKD5 vs. SAHA vs. TSA: 0.3 ± 0.41 μM vs. 9.3 ± 0.69 μM vs. 7.4 ± 0.86 μM, respectively, for SNU.GBM-2, and 0.4 ± 0.37 μM vs. 7.7 ± 0.40 μM vs. 52.1 ± 14.6 μM, respectively, for SNU.GBM-4, Supplementary Table S1). In cell proliferation assays, only CKD5 showed consistent anti-proliferative effects in all tested cell lines, even at the lowest concentration of 0.5 μM (Figure 1B). Next, we evaluated whether the anti-cancer effects of CKD5 were associated with apoptosis. With an exposure time of 48 h of CKD5, early apoptosis was significantly increased by approximately 1.6- to 4-fold in all glioblastoma cells (Figure 1C, Supplementary Table S2). We did not observe any increase in apoptosis in SNU.GBM-4 cells treated with TSA and in all cells treated with SAHA. Our results indicate that CKD5 was the most effective anti-cancer drug for glioblastoma cells among the tested pan-HDACIs.

### CKD5 strongly induces cell cycle arrest mediated by p21, CDK4 and CCND1

**Figure 2:**
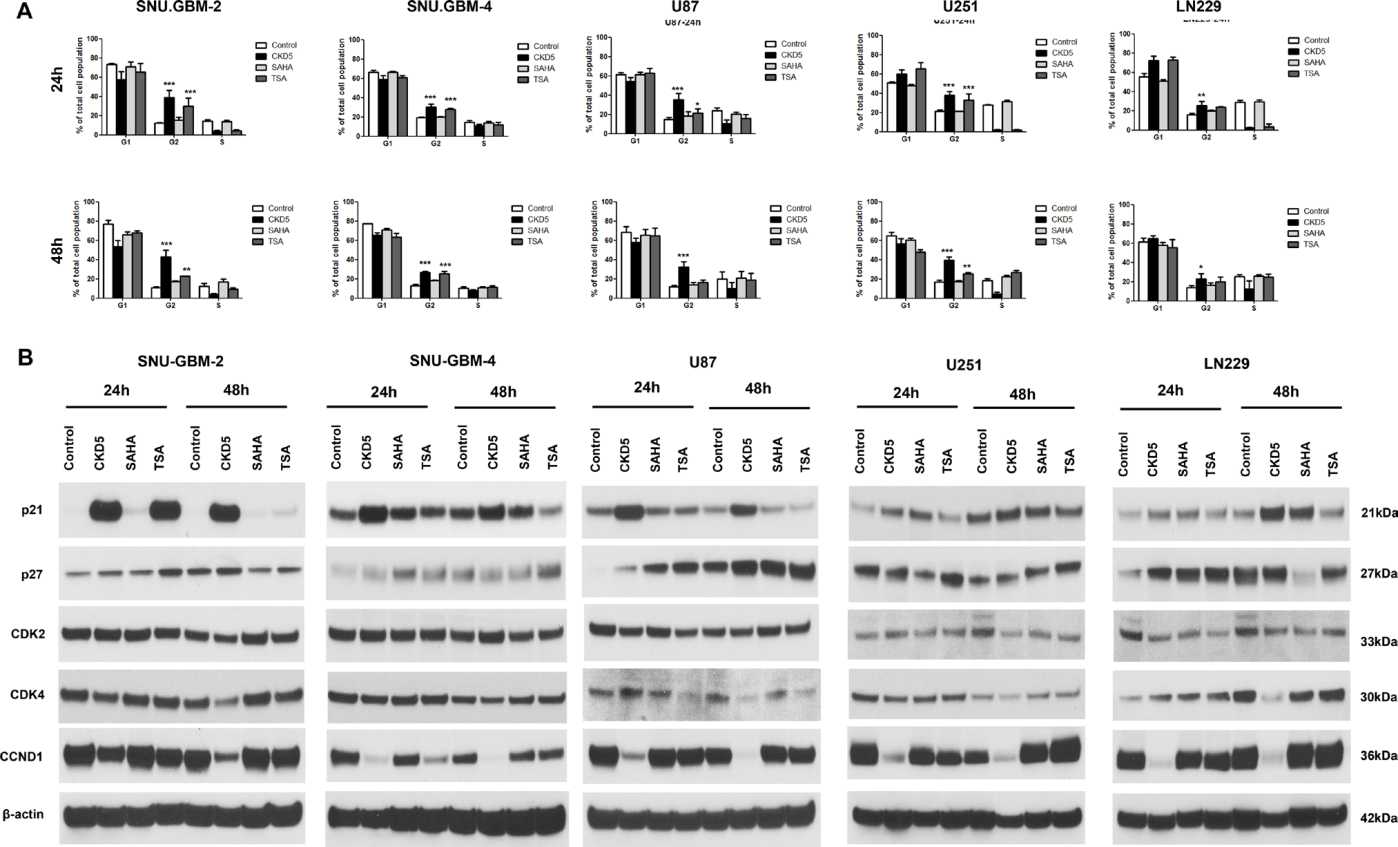
Cell cycle analysis and expression of cell cycle regulators Cell cycle distributions in glioblastoma cells with HDACI treatment. The analysis of cell cycle arrest in glioblastoma cells showed that the percentage of cells in G2-M phase is induced by 1.5- to 42.1-fold by CKD5 at 48 h. *p < 0.05, **p < 0.01, ***p < 0.005.

The effect of CKD5 on the cell cycle profile was analyzed by flow cytometry. CKD5 induced significant accumulation of the cells in G2 phase and led to a concomitant decrease in the population of cells in G1 and S phase in all glioblastoma cells after 24 h compared with SAHA and TSA (Figure 2A and Supplementary Table S3). We found that CKD5 can induce apoptosis-specific DNA fragmentation following induction of G2 arrest in glioblastoma cells. The percentage of cells in G2 phase increased by 1.5- to 42.1-fold following CKD5 treatment at 48 h.

Next, we investigated the molecular mechanism of cell cycle arrest by CKD5 by analyzing cell cycle related-proteins, such as p21, p27, CDK2, CDK4 and CCND1, with western blot analysis. There was a significant increase in expression of p21, and this was tightly linked to the reduction in CDK4 and CCND1 in all glioblastoma cells after CKD5 treatment (Figure 2B). This phenomenon was not found in cells after treatment of SAHA and TSA. To further explore the molecular mechanisms associated with the cell cycle arrest, we monitored expression of p27 and CDK2. However, there was no consistent pattern of changes in the levels of p27 and CDK2 in the glioblastoma cells. Overall, it is noteworthy that CKD5 was the most powerful regulator of the cell cycle, and its possible mediators are p21, CDK4 and CCND1.

### CKD5 is a more effective HDACI than SAHA and TSA

**Figure 3:**
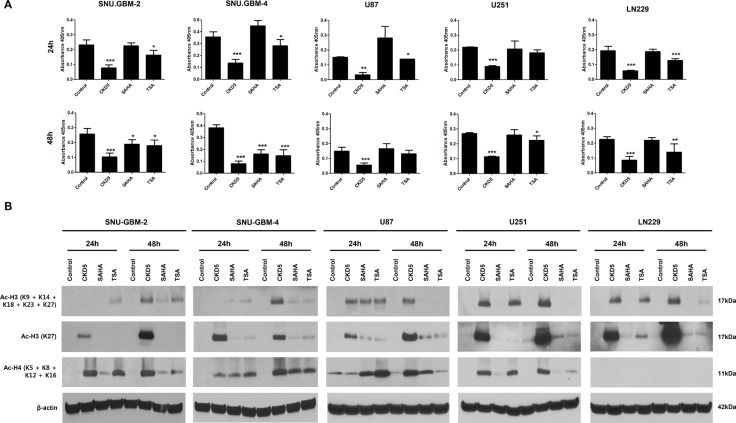
Histone deacetylase (HDAC) enzyme activity, histone H3 and H4 acetylation by HDACIs (**A**) CKD5 strongly decreases the enzyme activities by approximately 6- to 8-fold at 24 h, which was stable at 48 h in all glioblastoma cells. (**B**) CKD5 induces the acetylation status of histone H3 (Ac-H3) at 24 and 48 h.

To determine whether CKD5 efficiently inhibits HDAC enzyme activities, total HDAC enzyme activities were analyzed in different glioblastoma cells after treatment with CKD5, SAHA, and TSA at IC_50_ doses. CKD5 more significantly decreased the enzyme activities by approximately 6- to 8-fold at 24 h compared to SAHA and TSA, and it showed sustained inhibition at 48 h in all glioblastoma cells (Figure 3A). Additionally, we examined the acetylation status of histone H3 (Ac-H3) after 24 and 48 h. CKD5 more effectively induced histone H3 acetylation in all glioblastoma cells (Figure 3B)

### CKD5 effectively reduces the tumor volume in an orthotopic xenograft glioblastoma mouse model

**Figure 4:**
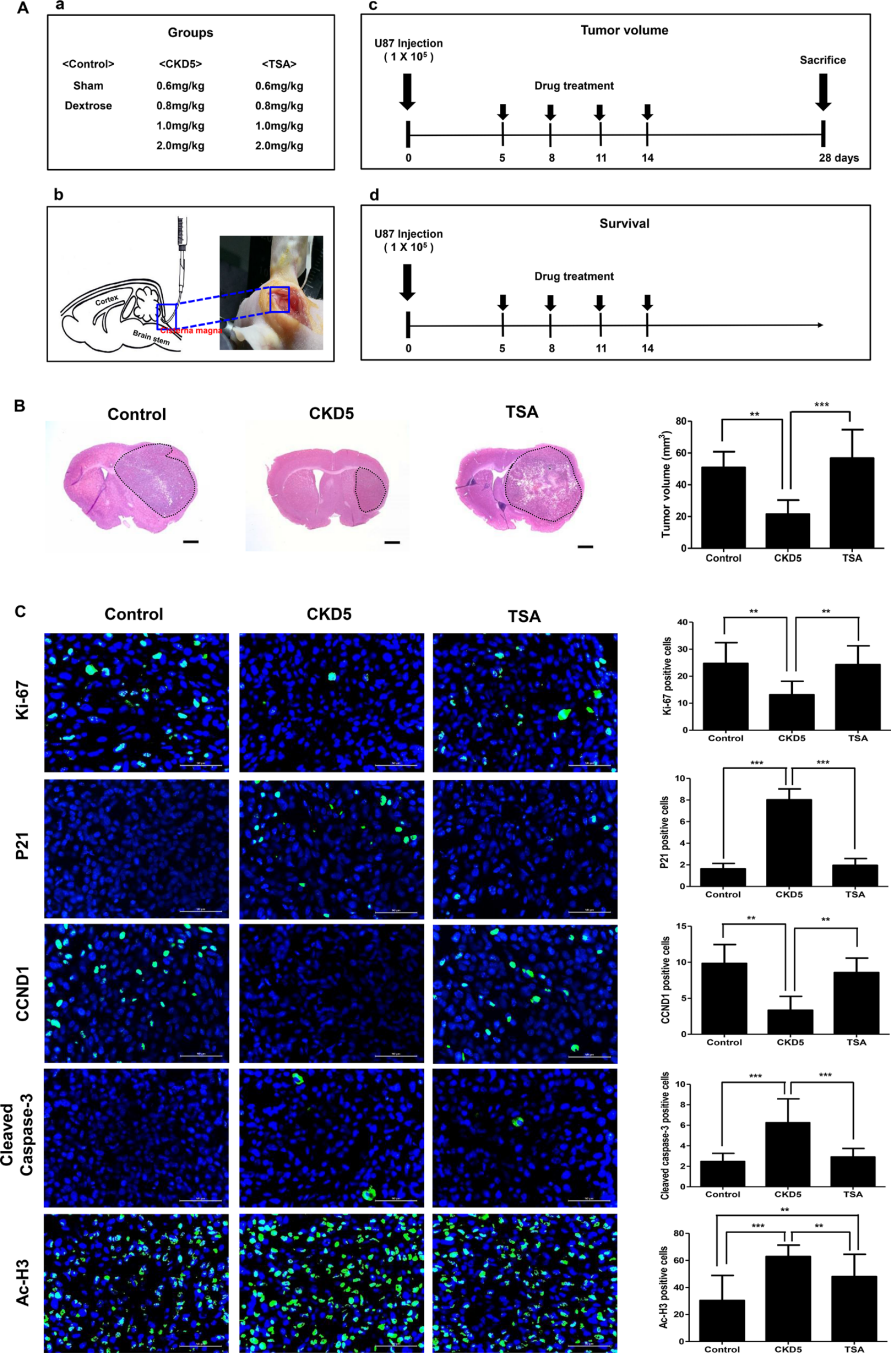
Short-term therapeutic efficacy of CKD5 CKD5 reduces tumor growth and prolongs survival rate in an orthotopic xenograft glioblastoma mice model. (**A**) Schematic plot of the study design and route of injection for short-term and long-term therapeutic efficacy. (**B**) Representative histological images show a 57% reduction in tumor volume by CKD5 (21.5 ± 8.9 mm^3^) compared with the control (50.9 ± 9.9 mm^3^, *p* < 0.01) or TSA (60.9 ± 9.2 mm^3^, *p* < 0.001). Hematoxylin and eosin (H&E) staining. Magnification, ×1.25. (**C**) Representative immunofluorescence images show Ki-67, p21, CCND1, cleaved caspase-3 and Ac-H3. Positive cells are shown in green. The graph indicates the number of positive cells compared with the control. Scale bar, 50 μm. Cells were counterstained with DAPI (blue). *p < 0.05, **p < 0.01, ***p < 0.005.

We confirmed the superior anti-cancer effects of CKD5 by *in vivo* experiments using an orthotopic xenograft glioblastoma mouse model. The overall design of the study, treatment groups, route of injection, and short-term/long-term treatment schedule are described in Figure 4A. We performed a pilot study to determine the optimal dosage of CKD5 (Supplementary Figure S1 and Supplementary Table S4). We found that two mice died after 0.8 mg/kg of CKD5 treatment. At high doses (1 and 2 mg/kg), CKD5 reduced the tumor volume by 70%, but toxic effects were observed. However, TSA had no therapeutic effect at any dose.

We compared the therapeutic effect of 0.8 mg/kg CKD5 with 0.8 mg/kg TSA and found that CKD5 showed stronger anti-cancer effects than SAHA *in vitro*. Cisternal injection of CKD5 significantly suppressed the tumor growth compared to the control (21.5 ± 8.9 mm^3^ vs. 50.9 ± 9.9 mm^3^, respectively, *p* < 0.01) or TSA (50.9 ± 9.9 mm^3^, *p* < 0.001, Figure 4B). There was no statistically significant difference between the control group and TSA-treated group in tumor volume (*p* > 0.05). Body weight monitoring of mice revealed that there were initial reductions in weight associated with HDACI administration. The reduction in weight continued until day 11 of treatment, remained stable through the treatment period, and was rapidly increased after completion of the treatment (Supplementary Figure S1). In the tumor samples, we found significantly increased p21, cleaved caspase-3 and Ac-H3 levels, with decreased numbers of cells that were positive for Ki-67 and CCND1 (Figure 4C).

### CKD5 effectively prolongs the survival in the orthotopic xenograft glioblastoma mouse model

**Figure 5:**
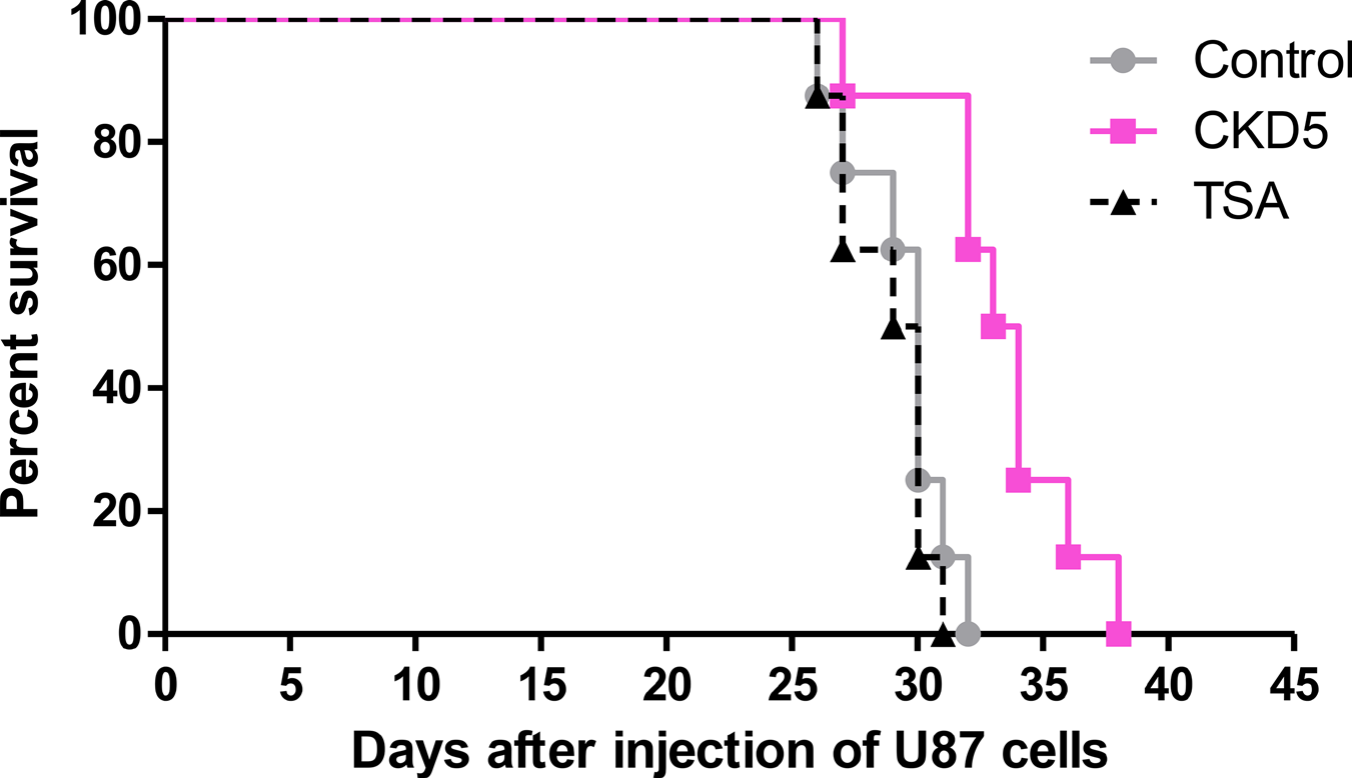
Long-term therapeutic efficacy of CKD5 Kaplan-Meier curves show that CKD5 prolongs the median survival of mice (34 days) compared with the control (30 days, *p* = 0.0024) or TSA (29 days, *p* = 0.0009) (control vs. CKD5 vs. TSA: 30 vs. 34 vs. 29 days, respectively).

For the survival analysis, we employed a separate set of experimental mice with orthotopic xenografts. A Kaplan-Meier survival curve showed a significant increase in survival in CKD5-treated mice compared with the control (median survival 34 days vs 30 days, respectively, *p* = 0.0024) and TSA (median survival 29 days, *p* = 0.0009, Figure 5). There was no statistically significant difference between survival of the control group and the TSA-treated group (*p* = 0.68). Taken together, our results suggest that CKD5 is a promising novel therapeutic agent for glioblastoma treatment.

## DISCUSSION

Among the variety of HDACIs being developed as therapeutics, HDACI hydroxamate derivatives targeting multiple Zn^2+^-dependent HDACs, so-called pan-HDACIs, have advanced the furthest in clinical application as an anti-cancer drug. SAHA was approved in 2006 by the US Food and Drug Administration for the treatment of cutaneous T-cell lymphoma [24]. SAHA is known to inhibit HDACs 1, 2, 3, and 6 [8]. TSA is another pan-HDACI similar to SAHA that inhibits HDACs 1, 2, 3, 4, 6, 7, and 10 and showed potent anti-cancer effects in pre-clinical experiments [8, 25–27]. There are pre-clinical studies reporting encouraging results for both SAHA and TSA in the treatment of glioblastomas [18, 19, 28–31]. However, clinical trials showed that the effect was modest, and it is recommended to use HDACIs with other cytotoxic drugs for treating glioblastomas [32, 33]. There were several limitations in the use of SAHA for glioblastoma therapy. The activity of SAHA in the brain is weak due to its short half-life and low blood-brain barrier (BBB) permeability; the BBB permeability-surface area product to free drug of SAHA is less than 2 orders of magnitude of that predicted by passive diffusion [8, 34]. The solubility of SAHA is very low in water (0.0716 mg/ml). In terms of safety issues, SAHA has cardiac toxicity, such as QTc prolongation [35], and is positive in the Ames test [36], indicating that it induces DNA point mutations and genotoxicity. Compared to SAHA, CKD5 has improved drug-like properties. The basic tertiary amine structure of CKD5 allows the formation of salts, which improves its water solubility (> 1,000 mg/ml). Furthermore, CKD5 is negative in the Ames test, and CKD5 showed no cardiac toxicity in pre-clinical and GLP studies. Taken together, these data suggests that CKD5 has a wider therapeutic window than SAHA.

In the present study, we demonstrated more potent anti-cancer effects of a novel pan-HDACI, CKD5, than SAHA and TSA at a lower dose in glioblastoma. The general molecular mechanisms of HDACIs include cell cycle arrest, induction of apoptosis, and downregulation of survival signals. The mechanism of action of CKD5 is similar to that of the other pan-HDACIs, such as SAHA and TSA. Thus, we focused on the evaluation of its effect on the cell cycle, apoptosis and survival-related protein expression. We found that CKD5 strongly increased p21 protein expression, which was correlated with growth inhibition, whereas TSA-induced growth inhibition was p21-independent. Regulation of p21 by SAHA was cell-type dependent. As the G2 population increased only after 24 h of CKD5 treatment in all cells, induction of apoptosis was accelerated with rapid cell cycle arrest at an earlier stage. This result was consistent with patient-derived glioblastoma cells, which showed the greatest sensitivity to CKD5; inhibition of cell growth was the highest in patient-derived glioblastoma cells. Additionally, p21 levels were the highest, and CDK4 and CCND1 levels were the lowest in these cells.

For therapeutic use of CKD5 in glioblastoma, delivery methods and dose of CKD5 should be titrated. CKD5 was administered intracisternally because it does not penetrate the BBB. We determined the effective concentration of CKD5 (Supplementary Figure S1 and Supplementary Table S4). Although CKD5 had powerful therapeutic effects at more than 0.8 mg/kg, this dosage resulted in serious toxicity. Therefore, we used the 0.8 mg/kg concentration and confirmed the reduction in tumor volume and the increase in survival in an orthotopic xenograft glioblastoma mouse model. Fine tuning of the optimal biological and chemotherapy scheduling is needed to maximize therapeutic efficacy and safety for clinical application of CKD5. The severe toxicity of the other pan-HDACIs, such as thrombocytopenia, and severe fatigue significantly limited their clinical use [37, 38]. CKD5 has a better safety profile and a wider therapeutic index compared to SAHA. Therefore, CKD5 may be used at a higher dose to reach the clinically effective range in cancer patients. To minimize the toxicity, CKD5 may be used as a therapeutic agent in combination with other drugs such as temozolomide or radiation for further improvement of glioblastoma therapy [39]. Confirmation of the effect for monotherapy is a priority before testing the combined therapy.

In this study, we demonstrated for the first time that a novel HDACI, CKD5, has strong anti-cancer effects with more susceptible patient-derived glioblastoma cells *in vitro*. We also confirmed that CKD5 was more powerful than TSA *in vivo*. Taken together, our results suggest that CKD5 can be used for improvement of the therapeutic strategy for glioblastoma.

## MATERIALS AND METHODS

### Isolation of glioblastoma tumor cells and cell culture

Glioblastoma tissue samples (World Health Organization Grade IV) were obtained from two patients (a 64-year-old man: *MGMT* promoter methylation, SNU.GBM-2 and a 55-year-old woman: *MGMT* promoter unmethylation, SNU.GBM-4) who underwent surgical excision at the Seoul National University Hospital and provided written informed consent approved by the Institutional Review Board (IRB # 1410-033-616). The tumor cells were isolated immediately after surgical resection and cultured as reported previously [24]. Only the cells that were under 4 passages in primary culture were used in this study. Human glioblastoma cell lines (U87, U251 and LN229) were purchased from the American Tissue Culture Collection (ATCC, Manassas, VA). All cells were cultured in Dulbecco's modified Eagle's medium (DMEM; WelGENE, Seoul, Korea) supplemented with 10% fetal bovine serum (FBS; Gibco, Grand Island, NY) and 1% antibiotics/antimycotics and incubated at 37°C in a humidified atmosphere of 5% CO_2_.

### HDAC inhibitors

CKD5 was obtained from Chong Kun Dang Research Institute, CKD Pharmaceuticals (Korea). It is a hydroxamic acid-based small molecule that chelates Zn in the active site of HDAC enzymes. The structure of HDACIs is composed of three parts (Zn binder, a linker and a CAP group). A novel pan-HDACI, CKD5 is structurally different from other pan-HDACIs, such as SAHA and TSA. All three compounds have the same Zn-chelating moiety, hydroxamate, but CKD5 has an aliphatic linker and urea for the CAP group. SAHA and TSA were purchased from Sigma-Aldrich (St. Louis, MO, USA). All inhibitors were dissolved in dimethyl sulfoxide (Sigma Aldrich) to concentrations of 10 mM and stored at −80°C.

### Cell viability and proliferation assay

Cell viability was assessed using the Cell Counting Kit-8 (CCK-8; Dojindo, Japan). Briefly, cells (4×10^3^ cells/well, SNU.GBM-2 and SNU.GBM-4 or 2×10^3^ cells/well, U87, U251 and LN229) were plated in each well of a 96-well plate and exposed to different concentrations of HDACIs for 24, 48 and 72 h. The relative cell viability (%) was calculated using the equation OD^T^/OD^C^×100% (where OD^T^ represents the absorbance of the treatment group, and OD^C^ represents the absorbance of the control group) as reported previously [24]. The median inhibitory concentration (IC_50_) was defined as the drug concentration required to inhibit 50% of the cells relative to the controls. IC_50_ values were estimated from the dose-response curve. For further studies, the IC_50_ values for CKD5, SAHA and TSA were used for 72 h (Supplementary Table S1).

### Apoptosis assay

Apoptotic cells were determined by annexin V-FITC and propidium iodide (PI) staining with a FITC-Annexin V apoptosis detection kit (BD Biosciences, San Jose, CA) according to the manufacturer's instructions. The stained cells were then analyzed by FACS (BD), and the results were analyzed by CellQuest software (BD).

### Cell cycle analysis

Cells (2 × 10^6^ cells/well, SNU.GBM-2 and SNU.GBM-4 or 1 × 10^6^ cells/well, U87, U251 and LN229) were plated in 100 mm plates and treated with IC_50_ values of the chemicals. After HDACI treatment with the given concentrations, cells were fixed using ice-cold 70% ethanol, washed with 1 × PBS and then suspended in propidium iodide (10 μg/ml) and ribonuclease A (0.1%). Cells were incubated for 30 min in the dark at room temperature. Propidium fluorescence was quantified after laser excitation of the fluorescent dye by FACS (BD) with a cell count of 10,000 cells per sample. Finally, DNA content of the cells in different phases of the cell cycle was determined using CellQuest Software (BD). We used synchronized cell population to perform the cell cycle and western blot analysis.

### Western blot analysis

After treatment with HDACIs, the cells were lysed with lysis buffer (Cell Signaling, Danvers, MA) including a protease inhibitor cocktail (Roche Diagnostics GmbH, Mannheim, Germany). Western blot analysis was performed as previously reported [40]. Antibodies used for immunodetection were anti-p21 (1:1,000, Abcam, Cambridge, MA), anti-p27 (1:500, Abcam), anti-CDK2 (1:500, Thermo Scientific, Rockford, IL), anti-CDK4 (1:200, Abcam), anti-acetyl H3 (K9 + K14 + K18 + K23 + K27, 1:1,000, Abcam), anti-acetyl H3 (K27,1:2,000, Abcam), anti-acetyl H4 (K5 + K8 + K12 + K16,1:2,000, Abcam) and anti-β-actin (1:10,000, Sigma-Aldrich).

After the blot was washed, it was incubated with a horseradish peroxidase-conjugated species-specific secondary antibody (1:5,000, Jackson Laboratory, West Grove, PA) for 1 h at room temperature. The blots were developed using a chemiluminescence detection system (Invitrogen, Carlsbad, CA). Band density was analyzed using NIH ImageJ software. Densitometric measurements were performed on individual immunoblot for each antibody tested, and the values indicate the protein level normalized to the corresponding β-actin levels.

### HDAC enzyme activity assay

Total HDAC enzyme activity was determined by the HDAC activity colorimetric assay kit (Biovision, Mountain View, CA) following the manufacturer's protocol. The cells were treated with HDACIs and collected using lysis buffer (Cell Signaling, Danvers, MA). The protein concentration was determined with the BCA assay (Pierce, Rockford, IL). Equal amounts of proteins (30 μg) were analyzed at 405 nm.

### *In vivo* short-term and long-term therapeutic efficacy

Animal experiments were carried out using a protocol approved by the animal facility of the Seoul National Institutional Animal Care and Use Committee (IACUC # 13-0363-C1A0) in accordance with the Guidelines for the Care and Use of Laboratory Animals published by the National Institutes of Health. Seven-week-old female BALB/c nude mice were purchased from OrientBio (Seongnam, Korea). The mice were anesthetized by intraperitoneal injection of a solution of 30 mg/kg zoletil (Virbac) and 10 mg/kg xylazine (Bayer Korea). U87 cells (1 × 10^5^) were injected stereotactically into the brains using a stereotactic device (1 mm anterior and 2 mm lateral to the bregma, 3 mm depth from the dura).

Five days after U87 tumor implantation, intracisternal injection of HDACIs was applied [41, 42]. Mice were randomly divided into three groups: the control, CKD5 or TSA treatment group (*N* = 8–11 per group). The groups were administered various concentrations (0.6, 0.8, 1.0 or 2.0 mg/kg) of CKD5 or TSA dissolved in 0.5% dextrose buffer every three days for two weeks. The mouse intrathecal catheter (32G, ALZET Osmotic Pumps, Cupertino, CA) was prepared as described previously [41] and inserted into a microsyringe (10 μl; Hamilton Company, NV). The infusion pump (Harvard Apparatus) was connected to the microsyringe. After anesthesia, the mice were secured in a stereotaxic frame, and the cisterna magna was punctured with a 30G needle to drain the cerebrospinal fluid. Then, the prepared catheter was inserted into the cisterna magna. HDACIs were slowly delivered in 5 μl volume at a rate of 1 μl/min. In control mice (*N* = 8), the cisterna magna was punctured and injected with only 0.5% dextrose.

To confirm short-term therapeutic efficacy, the mice were sacrificed after 28 days, and tumor volumes were measured as described previously [40]. To assess long-term therapeutic efficacy, mouse survival was measured as described previously [40]. Discomfort or distress was assessed by animal care personnel with no knowledge of the protocol design. All euthanized animals were verified as bearing tumors by necropsy.

### Immunofluorescence staining

After sacrifice, the mice were perfused, and the whole brain was fixed in 4% paraformaldehyde and embedded in OCT compound with 10 μm sections. Hematoxylin-eosin staining and immunofluorescence with antibodies against anti-Ki67 (1:200, Abcam), anti-p21 (1:400, Cell Signaling), anti-CCND1 (1:100, Thermo Scientific), anti-cleaved caspase-3 (1:200, Abcam), and anti-acetyl (Ac)-H3 (1:200, Abcam) were performed. The number of positive cells was counted in randomly taken pictures (5 pictures from each tumor, 8 tumors in each experimental group) by a confocal microscope and graphed.

### Statistical analysis

Statistical analysis of the data was performed using GraphPad Prism version 4.00 (GraphPad Software, San Diego, CA). All the experiments were performed more than three times to confirm the significance. To compare the mean variables among the groups and procedures, one-way or two-way ANOVA was used. Group means were compared by a Bonferroni's post -test. A Kaplan-Meier curve was used for survival analysis. *p* < 0.05 was considered statistically significant.

## SUPPLEMENTARY TABLES AND FIGURE


