# Supplementary material for: A novel histone deacetylase inhibitor, CKD5, has potent anti-cancer effects in glioblastoma
Source: Oncotarget. 2016 Nov 10;8(6):9123–33. doi: 10.18632/oncotarget.13265 (PMC5354719; doi:10.18632/oncotarget.13265)
Supplement: Supplementary file 1 [file oncotarget-08-9123-s001.pdf]

A novel histone deacetylase inhibitor, CKD5, has potent anti-cancer effects in glioblastoma

Supplementary Materials

Supplementary Table S1: Comparison of the 50% inhibitory concentration (IC50) for inhibitors in different glioblastoma cells

Groups	Hours	SNU.GBM-2	SNU.GBM-4	U87	U251	LN229
CKD5	24 h	12.3 ± 0.07	27.8 ± 9.22	62.3 ± 9.29	9.6 ± 0.73	1.7 ± 1.43
	48 h	10.6 ± 0.29	7.2 ± 3.47	11.5 ± 1.01	0.2 ± 0.33	0.9 ± 0.73
	72 h	0.3 ± 0.41	0.4 ± 0.37	3.9 ± 1.74	0.2 ± 0.36	0.2 ± 0.11
SAHA	24 h	–	–	–	8.0 ± 0.17	16.8 ± 2.75
	48 h	12.4 ± 0.79	9.7 ± 0.64	13.7 ± 1.23	7.9 ± 0.43	6.1 ± 0.15
	72 h	9.3 ± 0.69	7.7 ± 0.40	15.8 ± 2.77	6.2 ± 0.22	5.7 ± 0.25
TSA	24 h	–	–	15.8 ± 1.79	3.9 ± 0.29	0.8 ± 0.11
	48 h	–	35.1 ± 2.27	0.8 ± 0.15	0.6 ± 0.06	0.3 ± 0.03
	72 h	7.4 ± 0.86	52.1 ± 14.6	0.7 ± 0.01	0.5 ± 0.03	0.3 ± 0.01

Abbreviations: CKD5, 7-ureido-N-hydroxyheptanamide derivative: SAHA, suberoylanilide hydroxamic acid: TSA, Trichostatin A.
The IC50 value was calculated as the mean ± standard deviation (μM).

Supplementary Table S2: The proportion of early or late apoptotic cells in glioblastoma (%)

Apoptosis	Groups	SNU.GBM-2		SNU.GBM-4		U87		U251		LN229	
		24 h	48h	24 h	48 h	24 h	48 h	24 h	48 h	24 h	48 h
Early	Control	1.03 ± 0.50	0.99 ± 0.68	7.90 ± 1.61	9.99 ± 1.16	2.45 ± 1.83	2.07 ± 1.06	0.26 ± 0.21	0.25 ± 0.03	0.43 ± 0.29	0.21 ± 0.22
	CKD5	0.95 ± 0.33	2.69 ± 0.42***	8.56 ± 1.79	14.87 ± 0.42**	12.17 ± 2.28***	21.43 ± 3.49***	1.15 ± 0.21	7.51 ± 0.89***	2.74 ± 0.63*	8.28 ± 3.07***
	SAHA	0.66 ± 0.21	0.93 ± 0.42	11.8 ± 4.69	10.86 ± 1.32	3.37 ± 1.61	3.01 ± 1.71	0.18 ± 0.10	0.33 ± 0.11	0.64 ± 0.32	0.23 ± 0.79
	TSA	1.27 ± 0.11	1.79 ± 0.26	9.55 ± 0.55	11.36 ± 1.94	6.45 ± 0.18**	5.73 ± 3.60	0.76 ± 0.39	0.89 ± 0.21	1.65 ± 0.26	1.12 ± 0.42
Late	Control	5.22 ± 0.63	6.04 ± 0.68	7.86 ± 5.03	8.61 ± 3.07	3.21 ± 0.73	5.33 ± 2.90	2.57 ± 1.38	4.29 ± 3.14	3.85 ± 2.58	2.25 ± 0.82
	CKD5	4.82 ± 0.64	7.01 ± 0.79	7.13 ± 2.27	9.48 ± 1.91	4.59 ± 1.92	12.23 ± 1.57	5.99 ± 1.88	5.53 ± 1.78	4.65 ± 1.34	9.30 ± 5.09
	SAHA	4.21 ± 1.64	5.74 ± 0.26	7.48 ± 3.47	9.84 ± 2.48	3.45 ± 1.58	4.89 ± 2.41	2.93 ± 0.96	2.92 ± 0.87	2.51 ± 0.39	3.23 ± 1.91
	TSA	5.74 ± 2.96	6.18 ± 1.01	6.76 ± 2.34	8.21 ± 0.96	4.29 ± 1.43	5.98 ± 2.38	3.33 ± 0.23	2.88 ± 0.61	4.47 ± 1.38	3.83 ± 1.11

Abbreviations: CKD5, 7-ureido-N-hydroxyheptanamide derivative: SAHA, suberoylanilide hydroxamic acid: TSA, Trichostatin A.
Significantly different from control group (*$p < 0.05$, **$p < 0.01$, ***$p < 0.005$).

Supplementary Table S3: The phases of cell cycle (%)

phases	Groups	SNU.GBM-2		SNU.GBM-4		U87		U251		LN229	
		24 h	48 h	24 h	48 h	24 h	48 h	24 h	48h	24 h	48 h
G1	Control	73.09 ± 1.44	76.84 ± 3.93	66.49 ± 1.92	77.15 ± 0.35	61.49 ± 1.80	68.28 ± 6.11	50.64 ± 1.16	64.82 ± 3.29	55.19 ± 3.44	61.14 ± 4.21
	CKD5	57.51 ± 8.55	52.28 ± 6.44***	58.67 ± 3.92	65.44 ± 2.30**	54.46 ± 3.93***	57.66 ± 4.64	60.06 ± 4.27	56.46 ± 5.33	72.48 ± 4.13	64.73 ± 3.18
	SAHA	70.86 ± 4.73	65.79 ± 3.23	66.20 ± 1.02	70.72 ± 1.36	61.46 ± 2.39	65.16 ± 6.26	47.64 ± 1.69	60.35 ± 2.07	50.88 ± 1.55	57.72 ± 2.91
	TSA	65.45 ± 8.97	68.03 ± 1.64	60.68 ± 2.13	63.45 ± 4.07	62.92 ± 4.92**	65.71 ± 8.15	65.29 ± 6.34	47.64 ± 2.55	72.95 ± 3.04	55.44 ± 8.15
G2	Control	12.39 ± 0.23	10.91 ± 0.87	19.13 ± 0.76	12.65 ± 1.74	14.83 ± 2.11	11.87 ± 1.59	21.35 ± 1.32	16.79 ± 2.18	15.99 ± 1.52	13.74 ± 2.03
	CKD5	38.73 ± 7.54***	42.75 ± 7.11***	30.42 ± 2.78	26.71 ± 1.14***	35.14 ± 6.73***	32.26 ± 5.41***	38.07 ± 3.91***	39.12 ± 3.53***	25.39 ± 4.67**	22.87 ± 5.45*
	SAHA	15.39 ± 2.94	17.44 ± 0.40	19.92 ± 0.34	18.22 ± 0.49	18.35 ± 4.32*	13.79 ± 2.53	21.13 ± 0.12	17.11 ± 1.42	19.75 ± 1.33	16.24 ± 2.54
	TSA	30.04 ± 8.42***	16.76 ± 2.87***	11.68 ± 2.49	25.24 ± 2.77***	21.45 ± 4.17	16.28 ± 2.73	32.66 ± 6.46***	25.57 ± 1.15**	23.97 ± 0.52	19.73 ± 5.29
S	Control	14.52 ± 1.45	12.25 ± 3.07	14.38 ± 2.03	19.19 ± 1.87	23.68 ± 3.34	19.85 ± 7.51	28.03 ± 0.18	18.39 ± 1.80	28.82 ± 1.94	25.12 ± 2.21
	CKD5	3.76 ± 1.02	3.96 ± 0.73	10.90 ± 1.23	7.85 ± 1.16	10.39 ± 3.82	10.08 ± 6.36	1.87 ± 0.37	4.42 ± 2.04	2.14 ± 0.66	12.41 ± 8.63
	SAHA	13.76 ± 2.94	16.76 ± 2.87	13.88 ± 1.34	11.05 ± 0.92	20.19 ± 1.92	21.05 ± 7.01	31.23 ± 1.58	22.54 ± 1.50	29.37 ± 1.88	26.04 ± 1.10
	TSA	4.51 ± 0.90	9.16 ± 1.50	11.68 ± 2.49	11.31 ± 1.56	15.63 ± 4.08	19.01 ± 6.88	2.05 ± 0.23	26.79 ± 1.90	3.18 ± 3.21	24.82 ± 2.86

Abbreviations: CKD5, 7-ureido-N-hydroxyheptanamide derivative: SAHA, suberoylanilide hydroxamic acid: TSA, Trichostatin A.
Significantly different from control group (*$p < 0.05$, **$p < 0.01$, ***$p < 0.005$).

Supplementary Table S4: Anti-cancer effect of various histone deacetylase inhibitors at different doses

Groups	Test Item	Dose (mg/kg)	Survival rate (%)	Reduction of tumor volume (%)
Non-treated	Sham	0 µl	100 (8/8)	0
	Control (0.5% dextrose buffer)	5 µl	100 (8/8)	0
CKD5	CKD5-0.6	0.6	92 (9/11)	0
	CKD5-0.8	0.8	78 (8/11)	57
	CKD5-1.0	1.0	38 (3/8)	73
	CKD5-2.0	2.0	25 (2/8)	70
TSA	TSA-0.6	0.6	75 (6/8)	0
	TSA-0.8	0.8	100 (8/8)	0
	TSA-1.0	1.0	88 (7/8)	0
	TSA-2.0	2.0	75 (6/8)	0

Abbreviations: CKD5, 7-ureido-N-hydroxyheptanamide derivative: TSA, Trichostatin A.

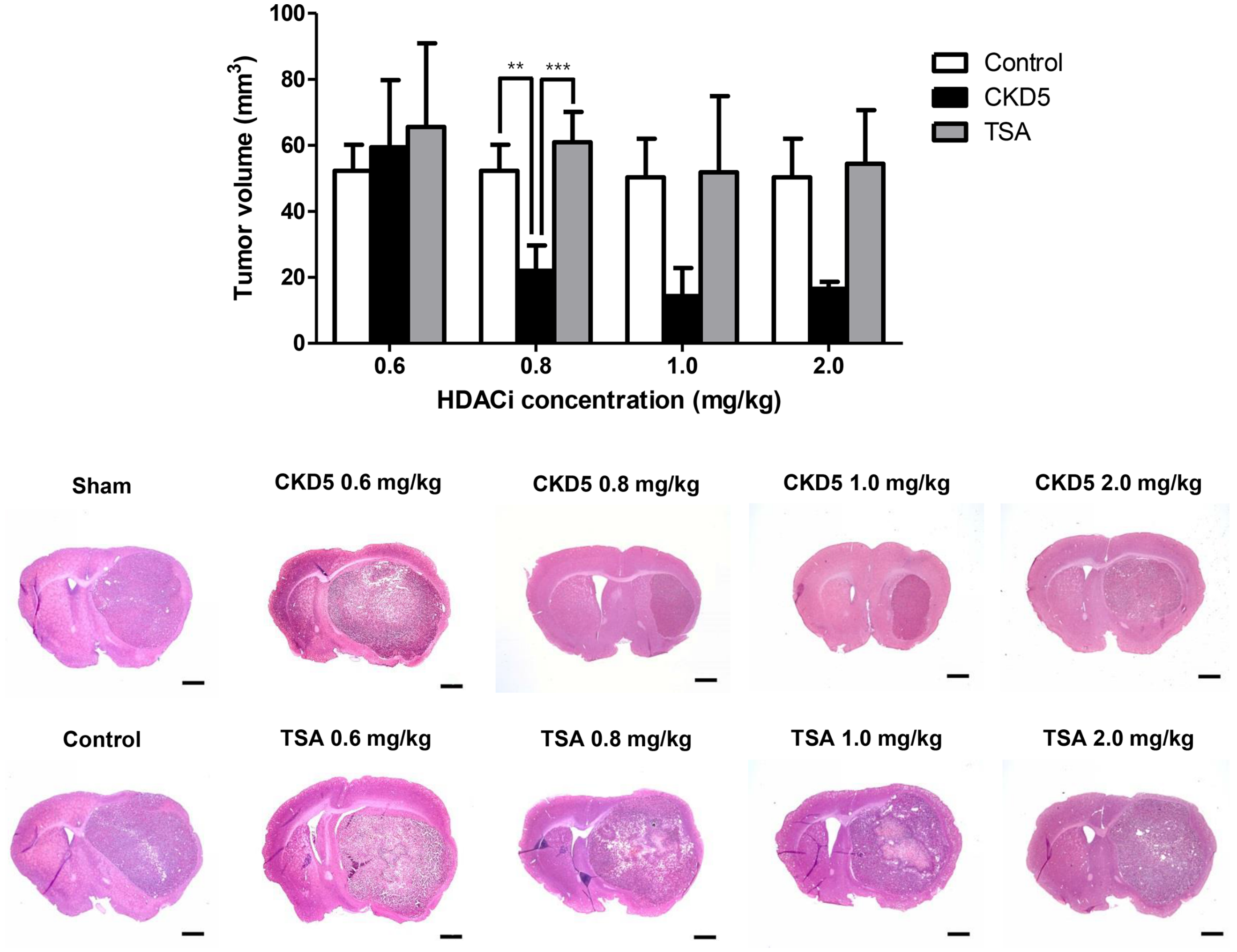

Supplementary Figure S1: Tumor volume by various histone deacetylase inhibitors (HDACIs) concentration. Tumor volume was examined after treatment with different concentrations of HDAC inhibitors. Tumor volume was reduced by 0.8, 1.0 and 2.0 mg/kg CKD5 but not TSA. Hematoxylin and eosin (H&E) staining. Magnification, ×1.25.
